# Exploring the impact of equipment modifications on novice tennis players: a scoping review

**DOI:** 10.3389/fpsyg.2025.1536427

**Published:** 2025-02-26

**Authors:** Ana Piquer-Piquer, Miguel Crespo, Jesús Ramón-Llin, José Francisco Guzmán, Rafael Martínez-Gallego

**Affiliations:** ^1^Department of Physical Education and Sport, University of Valencia, Valencia, Spain; ^2^Development Department, International Tennis Federation, London, United Kingdom; ^3^Department of Teaching of Music, Visual and Corporal Expression, University of Valencia, Valencia, Spain

**Keywords:** racquet sports, scaled equipment, skill acquisition, performance, psychological benefits, coaches’ perceptions, biomechanics

## Abstract

Tennis equipment modifications, such as smaller rackets and low-compression balls, are increasingly being used because they can better align with beginners’ physical capabilities, enhancing learning and engagement. This scoping review aimed to map current research on equipment modifications for beginner tennis players, identifying how these modifications impact skill acquisition, game performance, biomechanical variables, psychological aspects, and coaches’ perspectives. Searches across the Web of Science, Scopus, PubMed, and SPORTDiscus, along with expert input following the PRISMA procedures, yielded 35 studies. These studies involved empirical interventions related to scaled tennis equipment for beginners and were published in English or Spanish. Narrative reviews and studies lacking empirical data were excluded. The results indicate that equipment modifications enhance control, technique, and engagement in skill acquisition, improve tactical play with more aggressive strokes, and reduce joint stress, thereby minimizing injury risk. Psychological benefits include greater enjoyment and self-efficacy, and coaches strongly support these adaptations. These findings suggest practical implications for junior tennis development. However, future research should focus on expanding real-game applications, increasing participant diversity, and conducting detailed psychological and biomechanical assessments to further optimize player progression.

## 1 Introduction

The initiation phase in any sport is crucial for the comprehensive development of players as it establishes the foundation for their future athletic careers. In this context, sports equipment plays a pivotal role in shaping tactical understanding, technical learning, physical adaptation, and mental readiness during the initial performance of beginners. Contemporary motor learning theories, such as the Constraints-Led Approach (CLA), emphasize the importance of adjusting constraints related to the player, environment, and task to facilitate skill development (Davids et al., 2008). From this perspective, adapting sports equipment to suit the characteristics of beginner players has garnered growing interest in scientific research and sports practice.

The CLA approach supports the notion that the design of sports equipment and the dimensions of the playing area should be tailored to athletes’ physical needs, technical skills, and experience. Numerous studies have shown the benefits of such adaptations, particularly in enhancing skill acquisition, competitive performance, and enjoyment of the activity (Farrow and Reid, 2010; Buszard et al., 2014b; Kachel et al., 2015; Limpens et al., 2018). In tennis, initiatives such as Play and Stay and Tennis 10s, promoted by the International Tennis Federation (ITF), have implemented strategies involving the use of smaller rackets, low-compression balls, and scaled-down courts to adapt the sport to the needs of developing players. These strategies are organized into three progressive phases: red, orange, and green balls (International Tennis Federation, 2023).

Existing evidence highlights the positive impact of adapted equipment on tennis player development, particularly among children and young beginners. For example, (Buszard et al., 2020a) reported improvements in stroke accuracy, swing stability, and movement variability. Similarly, Larson and Guggenheimer (2013) noted enhancements in control, speed, and overall success, allowing beginner gameplay to more closely resemble adult gameplay using standard equipment. Beyond technical benefits, these adaptations also have psychological advantages, offering higher success rates and greater enjoyment, which promote skill learning in beginners (Farrow and Reid, 2010).

Despite the extensive literature in this field, existing reviews have focused on specific areas, such as adaptations in competitive contexts or skill acquisition processes, often addressing these topics in isolation (Buszard et al., 2016a; Chapelle et al., 2023). However, recent studies have identified complementary research lines and studies not included in previous reviews, underscoring the need for a more holistic perspective.

In this context, the present article aimed to review the existing literature on equipment modifications in tennis, differentiating key research lines and identifying knowledge gaps that could inform future studies. The central question guiding this review was as follows: What does the scientific literature reveal about scaled equipment in tennis and game adaptations concerning skill acquisition, match performance, biomechanical and psychological variables, and coaches’ perspectives? This comprehensive approach sought to provide an updated overview of the various dimensions of adapted equipment in tennis, fostering a broader and more multidimensional understanding of its impact on player development.

## 2 Methods

This scoping review was conducted following the protocol established by the Preferred Reporting Items for Systematic Reviews and Meta-Analysis Protocols (PRISMA-P), specifically adhering to the recommendations of the PRISMA Extension for Scoping Reviews 2020 (PRISMA-ScR) (Tricco et al., 2018).

### 2.1 Information sources

The literature search was conducted across different databases: the multidisciplinary Web of Science and Scopus; the biomedical and life sciences database PubMed; and the sport-dedicated database SPORTDiscus. Additional articles not indexed in these databases were provided by experts in the field. Grey literature sources were identified by searching titles through Google Scholar and the ITF Coaching & Sport Science Review journal, the official journal of the ITF. References from relevant articles were also reviewed to identify additional studies using the snowballing method (Naderifar et al., 2017).

### 2.2 Search strategy

The systematic search included all publications up to August 2024 to ensure a comprehensive identification of sources related to the topic. The following search terms and operators were applied across all databases in title-abstract-keywords parameters: (*tennis NOT table NOT elbow*) *AND* (scal* OR adapt* OR modif* OR mini*). These terms were selected after expert consultation. All database searches were performed by a single researcher to maintain consistency in the process.

### 2.3 Eligibility criteria

The criteria for article selection were established by the researchers following the PECOS framework (Higgins et al., 2019), as detailed in Tables 1, 2.

**Table 1:** Selection criteria for the scoping review.

Category	Inclusion criteria	Exclusion criteria
Type of study	Article, review article	Book, book chapter, systematic reviews
Language	English or Spanish	Other languages
Methodological design	Interventional or empirical	Reviews, without intervention

The focus of the review was on novice tennis players, so it was a priority to include young tennis players between the ages of 6 and 14 as participants. However, it was also considered important to include other participants without prior experience, such as beginner adult players or players with disabilities.

Regarding the exposure criteria, studies with a cross-sectional design (data collection lasting between 1 and 5 days) were included. These studies involved participants with no prior training under adapted conditions and only observed differences between different conditions. Studies involving an intervention (either short-term or long-term) were also considered, corresponding to a training period with adaptations. Long-term exposure was defined as a training duration of 5 weeks or more since the longest intervention observed lasted 8 months.

To be included in the review, the research had to relate to scaled or adapted equipment in the sport of tennis, specifically focusing on intervention-based or empirical studies. Eligible studies were required to include a data collection or measurement process, accompanied by data analysis—whether qualitative, quantitative, or mixed methods—concerning the use of adapted equipment in tennis training, match performance, coaches’ perspectives, or other relevant variables. In addition, this review was restricted to manuscripts written in Spanish or English due to the linguistic capabilities of the researchers and because these were the predominant languages in the literature. Consequently, narrative documents, reviews synthesizing articles already included in the study, and publications in languages other than Spanish or English were excluded.

### 2.4 Selection of the sources of evidence

Titles and abstracts were sequentially evaluated, and those unrelated to the main topic were discarded. After removing duplicates, the full text of the remaining publications was reviewed to ensure compliance with the inclusion criteria. If the exclusion criteria, listed in Table 2, were identified during the full-text reading, the article was excluded. All titles and abstracts were independently reviewed by two researchers. To ensure a rigorous process, only articles deemed eligible by both reviewers were included based on the inclusion criteria. In cases of discrepancies, discussions were held until consensus was reached. This procedure aimed to minimize bias in the study selection process. For the systematic reviews identified in the process, the number of studies potentially meeting the inclusion criteria was noted, particularly if those studies had been overlooked during the initial search.

**Table 2:** Inclusion and exclusion criteria for each PECOS domain in relation to adaptations in the players’ environment.

PECOS	Inclusion criteria	Exclusion criteria
Population	Tennis players under 10, 12, and 14 years old with or without experience. Players of all ages with disabilities. Adult novice tennis players. Tennis coaches and professional experts with experience.	Tennis players over 14 years old with experience. Adult players with experience. Tennis coaches without experience in adapted conditions.
Exposure	Transversal studies (less than a week of exposure). Short-term studies (1–4 weeks of training). Long-term studies (more than 5 weeks of training).	Without exposure to adapted conditions.
Comparison	Playing tennis with adapted conditions/materials compared to non-adapted conditions or standard materials.	
Outcome	Results regarding the influence of adaptations on physical, biomechanical, technical-tactical, or psychological variables. Opinions of coaches and experts on adapted equipment.	
Study design	Interventional or experimental quantitative studies. Selective studies (quantitative or qualitative). Observational studies.	Reviews and summaries without intervention. Articles where no original data were analyzed.

### 2.5 Critical appraisal of the individual sources of evidence

The quality of the articles included in the study was assessed using an adapted version of the Downs and Black (1998) checklist. Each article was evaluated on the following aspects: study purpose, background literature, study design, sample characteristics, statistical significance, data analysis methods, results, conclusions, and implications for future research (see footnote in Table 3). Each criterion was scored as follows: “+” if fulfilled, “–” if not fulfilled, and “NR” if the information was not reported. The scores were summed to obtain a total score, categorized as follows: less than 5 indicated low quality, between 5 and 7 indicated good quality, and 8 or more indicated high quality (van der Fels et al., 2015). The aim of this assessment was to identify and exclude any low-quality studies from the review.

**Table 3:** Quality assessment of the articles included (Downs and Black, 1998).

	Question number										
Author (year)	1	2	3	4	5	6	7	8	9	10	Total
Coldwells and Hare (1994)	+	−	+	NR	+	+	+	+	+	−	7
Hammond and Smith (2006)	+	+	+	+	+	+	+	+	+	+	10
Farrow and Reid (2010)	+	+	+	−	+	+	+	+	−	−	7
Hardoy et al. (2011)	+	−	+	+	+	+	+	+	−	+	8
Larson and Guggenheimer (2013)	+	+	+	+	+	+	+	+	+	+	10
Sánchez-Alcaraz (2013)	+	+	+	NR	+	+	+	+	−	−	7
Buszard et al. (2014a)	+	+	+	−	+	+	+	+	−	−	7
Buszard et al. (2014b)	+	+	+	+	+	+	+	+	+	−	9
Kachel et al. (2015)	+	+	+	NR	+	+	+	+	+	−	8
Timmerman et al. (2015)	+	+	+	NR	+	+	+	+	−	+	8
Buszard et al. (2016a)	+	+	+	+	+	+	+	+	+	+	10
Bayer et al. (2017)	+	+	+	+	+	+	+	+	−	−	8
Fitzpatrick et al. (2017)	+	+	+	+	+	+	+	+	+	+	10
Prodan and Grosu (2017)	+	+	+	+	+	+	NR	+	−	−	7
Sanz (2017)	+	+	−	−	+	−	+	+	−	−	5
Schmidhofer et al. (2014)	+	+	+	+	+	+	+	+	−	−	8
Ciuntea (2018)	+	+	+	NR	+	+	NR	+	−	−	6
Fitzpatrick et al. (2018)	+	+	+	+	+	+	+	+	+	−	9
Limpens et al. (2018)	+	+	+	+	+	+	+	+	+	+	10
Cortela et al. (2019)	+	+	+	NR	NR	−	NR	+	+	−	5
Davies (2019)	+	+	+	+	NR	−	NR	+	−	−	5
Giménez-Egido et al. (2020c)	+	+	+	+	+	+	+	+	−	−	8
Buszard et al. (2020c)	+	+	+	+	+	+	+	+	−	−	8
Buszard et al. (2020a)	+	+	+	+	+	+	+	+	+	+	10
Buszard et al. (2020b)	+	+	+	+	+	+	+	+	+	+	10
Gimenez-Egido et al. (2020a)	+	+	+	NR	+	+	+	+	+	+	9
Gimenez-Egido et al. (2020b)	+	+	+	+	+	+	+	+	+	−	9
Broadbent et al. (2021)	+	+	+	+	+	+	+	+	+	+	10
Fauzi et al. (2021)	+	+	+	+	+	+	+	+	+	+	10
Fadier et al. (2023)	+	+	+	+	+	+	+	+	+	+	10
Martínez-Gallego et al. (2022)	+	+	+	+	+	NR	+	+	+	+	9
Kilit et al. (2023)	+	+	+	NR	+	+	+	+	+	+	9
Touzard et al. (2023)	+	+	+	+	+	+	+	+	+	+	10
Gimenez-Egido et al. (2023)	+	+	+	NR	+	+	+	+	+	+	9
Kilit et al. (2024)	+	+	+	+	+	+	+	+	+	+	10

NR = not recorded; + = meets criteria; − = does not meet criteria. Questions: (1) Was the objective of the study clearly stated? (2) Was the relevant precedent literature reviewed? (3) Was the sample described in detail? (4) Was the sample size justified? (5) Was the design appropriate to the research question? (6) Were the results reported in terms of statistical significance? (7) Were the methods of analysis appropriate to the research design? (8) Were the conclusions appropriate given the findings of the study? (9) Are there implications for future research given the results of the study? (10) Did the authors acknowledge and describe the limitations of the study?

### 2.6 Data charting process

After the initial classification of the sources, a matrix was developed to extract the most relevant data from the included articles. The matrix captured the following:

1. Authors.
2. Year of publication.
3. Study objectives.
4. Sample and methodology.
5. Main results.

Using this information, the studies were categorized into the following emerging research lines:

- Skill acquisition and learning processes.
- Match performance.
- Influence on psychological variables.
- Analysis of biomechanical variables.
- Perceptions and opinions of coaches and professionals.

## 3 Results

### 3.1 Selection of the sources of evidence

**Figure 1:**
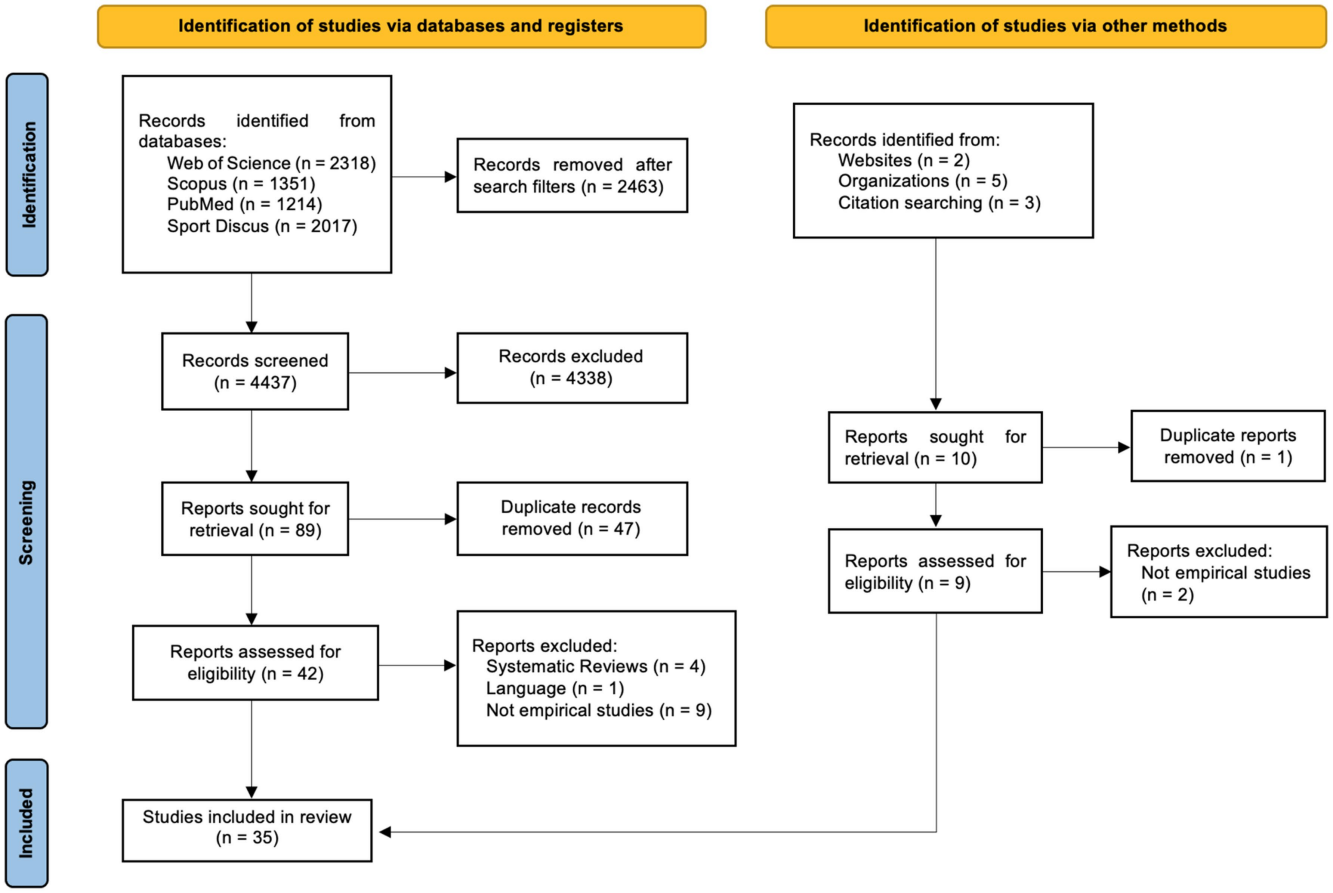
Flowchart based on the PRISMA guidelines.

Of a total of 6,900 articles identified, after the refinement procedure and application of the exclusion criteria, the final sample consisted of 35 studies. The selection and screening processes of the studies finally included in the review, according to the PRISMA procedure, are graphically presented in Figure 1.

### 3.2 Critical appraisal within the sources of evidence

Table 3 shows the quality scores for each article included in the scoping review, following the Downs and Black (1998) checklist. A total of 74% of the studies received high-quality ratings, and all exceeded the score of 5, at which the articles were considered to have good quality.

### 3.3 Characteristics of the sources of evidence

The main characteristics of each study included in this scoping review are summarized in Table 4. The full matrix detailing the characteristics of each study can be found in the Supplementary materials.

**Table 4:** Summary of the articles’ main characteristics.

Reference	Topic	Sample and methods	Results
Coldwells and Hare (1994)	MT skill transfer to real game.	*N* = 16 (7–10 years) in the EG / CG	MT showed better transfer of skill acquisition to real tennis than the CG.
Hammond and Smith (2006)	LCB in learning tennis skills.	*N* = 14 (5–11 years) in the EG/CG. 8 sessions.	LCB improved technique, length of points, and hitting opportunities.
Farrow and Reid (2010)	Adaptations on skill acquisition.	*N* = 23 (8 years without experience). 5 weeks.	Adapted conditions improved rally volume, technique, chances, success, enjoyment, and commitment.
Hardoy et al. (2011)	MT intellectual disabilities.	*N* = 24 (18–40 years) in the EG / CG. 6 months, 3h twice a week.	MT decreased the anxiety scale and improved coordination.
Larson and Guggenheimer (2013)	Modified court and ball on the forehand.	*N* = 8 (7–9 years with experience)	LCB and reduced courts resulted in higher speed, accuracy, and success scores.
Sánchez-Alcaraz (2013)	Adaptations over time structure and actions of play.	*N* = 8 (8 years with experience) 16 matches (8 orange, 8 yellow)	Orange court and ball: longer points, fewer unforced errors, and a greater number of strokes and winners.
Buszard et al. (2014a)	Examine racket sizes and ball compressions.	*N* = 80 (6–8 years without experience). Task with 3 conditions.	Small racket + red ball: the best hitting performance. Red ball: better with a small/medium racquet.
Buszard et al. (2014b)	Observe learning a motor task with scaled material.	*N* = 40 (9–11 years). Task with 2 conditions.	Adapted equipment: stepped forward, swung from bottom to top, correct impact with the ball more often.
Schmidhofer et al. (2014)	Differences in play structure between ATP and Tennis 10s.	*N* = 87 (67 elite players, 20 children).	U9: scores were better when playing on a smaller court.
Kachel et al. (2015)	Adaptations in junior competition.	*N* = 20 (10 years). 40 matches: standard/ green.	Modified ball: faster rally speed, more comfortable height shots, and at the net. Yellow ball: more “high” shots.
Timmerman et al. (2015)	Court sizes and net height in child tennis players.	*N* = 16 (9 years). 4 conditions combining a scaled court and net with the standard.	Scaled net: more winners, more forced errors, more shots at a comfortable height, and a more aggressive style of play.
Buszard et al. (2016b)	Scaled rackets on skill acquisition primary school.	*N* = 46 (Grade 1–2 PE). SR group, LR group. 30min x 5 weeks.	SR: Improved both the forehand and backhand and transferred the skills to the larger racket
Bayer et al. (2017)	Lime court in the transition from orange to green.	*N* = 24 (9–10 years) 12 matches on a green court and 12 on a lime court.	Lima: the structure of play more similar to adult game than the green court, more forced errors and winners, more points won with the 2nd serve, and ICT closer to an adult.
Fitzpatrick et al. (2017)	Court and ball dimensions over playing behavior.	*N* = 48 divided into 4 age groups (7, 8, 9, and 13 years). 35 matches.	Duration of points and number of forehand strokes declined; variability of play increased as age groups progressed.
Prodan and Grosu (2017)	Investigate racket sizes and ball compression on the backhand.	*N* = 20 (8–9 years) in the EG and CG. Red, orange, and green balls.	The accuracy-speed variable was much higher with scaled equipment. Low-pressure balls resulted in more successful shots.
Sanz (2017)	Compare traditional and adapted methodology.	*N* = 100 (7–10 years) in 2 EGs (orange & green ball) and 2 CGs.	The EG showed greater improvements than the CG, both at the technical and execution levels, as well as at the tactical level.
Ciuntea (2018)	Analyze Tennis 10s on the development of motor skills.	*N* = 56 (7–9 years) in the EG and CG. 8 months of 2 x sessions/week.	The EG improved more than the CG. Children up to 10 benefit from the Tennis 10s because of the gradual resizing of the materials.
Fitzpatrick et al. (2018)	Investigate adaptations in match behavior and skill tests.	*N* = 16 (7 years) in the EG and CG. 8-week intervention 1h/week.	EG: better stroke symmetry, more backhand strokes, and fewer forehand strokes. Higher backhand success rates, ability, and technical proficiency.
Limpens et al. (2018)	Examine reductions of net height on match performance.	*N* = 16 (9 years) 4 matches per pair with 4 net heights.	Lower net height: improved serving performance, more aggressive play, faster rallies, more winners, and decreased rally length.
Cortela et al. (2019)	Describe the transition from green to yellow ball.	*N* = 14 Brazilian club coordinators with experience in programs.	Not possible to identify criteria; the clinical eye of the coach is the main parameter for determining the transition.
Davies (2019)	Perspective of coaches on adapted equipment.	*N* = 20 coaches with experience in scaled equipment.	Emphasized that transitions should be based on skill levels rather than age to ensure adequate skill acquisition before moving to the next level...
Giménez-Egido et al. (2020c)	Impact of a modified competition on groundstrokes.	*N* = 20 (U10) 4 matches each, in different net heights and court lengths.	Modified competition: increased the number of flat shots and a more offensive style of play, but less variability in shots.
Buszard et al. (2020c)	Study different arm segments as determining variables.	*N* = 21 (6–9 years) in the EG and CG. 40 attempts at forehand.	Shoulder-racquet distance is a determining variable. Adapted equipment: more distal control in hand-racquet distance.
Buszard et al. (2020a)	Coaches’ perceptions of a modified tennis campaign.	*N* = 114 (35 key figures from national tennis associations and 79 coaches)	Perceived Play and Stay campaign increased and sustained participation, facilitated skill development, and had a positive effect on attitude.
Buszard et al. (2020b)	Adaptations over coordination and variability.	*N* = 25 Forehand hitting task using 2 pieces of equipment: scaled and normal.	Adapted materials: greater accuracy, temporal stability of the swing, and better coupling between the upper arm and forearm.
Gimenez-Egido et al. (2020a)	Compare 2 adapted competitions on serving in U10 players.	*N* = 20 (U10) 80 matches services, 40 in U10 and 40 in adapted conditions.	Modified competition: more effective serves, direct serves, unreturned serves, and improved serving performance.
Gimenez-Egido et al. (2020b)	Modifying net height and court length on technical-tactical.	*N* = 20 (U10) 40 matches, 20 U10 conditions, 20 adapted conditions.	Modified condition: more variability, shots close to the net, offensive play, and greater serve effectiveness (fewer 2nd serves).
Broadbent et al. (2021)	Calculating pi ratios for adaptations in junior sports.	Federation guidelines compared to children’s dimensions to calculate ratios.	Most children compete at larger sizes than would be appropriate, particularly younger ones. Girls’ competitions are better suited than boys’.
Fauzi et al. (2021)	Mini-tennis training on forehand groundstrokes.	*N* = 44 (6–8 years) 22 in the EG and 22 in the CG.	The EG performed better on the post-test than the CG. Significant differences were found between the pre-test and post-test.
Martínez-Gallego et al. (2022)	Find out experts’ opinions on Play and Stay	*N* = 35 experts from federations answered a questionnaire.	Most of them were aware of the rule change, applied it, had access to materials, and agreed it had a positive impact on U10 players.
Fadier et al. (2023)	Influence of serve distance and net height on biomechanics.	*N* = 10 (9–12 years) 3 serves from 3 distances (red, orange, and green courts).	Red conditions: more powerful, faster serves, improved maximum angular velocities of trunk and knee flexion.
Kilit et al. (2023)	Ball and court adaptations on skill learning in adults.	*N* = 24 university students. 6-week training.	LCB group: higher accuracy, rally performance, technique control, speed, direction, greater stability, and pace of play.
Touzard et al. (2023)	Racket size on serve biomechanics and performance.	*N* = 9 (10 years) 3 serves, 3 racquet sizes.	Racket size: not influenced ball speed or racket head speed. LR: increased shoulder and elbow loads, raising the risk of upper limb injury.
Gimenez-Egido et al. (2023)	Net height and court size on self-efficacy and efficiency.	*N* = 20. Assessed Perceived Physical Ability + Self-Efficacy.	Reduced net height and court size: higher self-efficacy and service efficiency.
Kilit et al. (2024)	LCB versus standard balls in adults.	*N* = 24 (18–34 years). 24 matches. Psychophysiological state and match play.	Green ball: higher HR, % HR, and enjoyment, longer rallies, more controlled pace, and lower perceived and mental efforts.

EG = experimental group; CG = control group; MT = mini tennis; ATP = Association of Tennis Professionals; LCB = low compression balls; U10 = under 10 years old players; SR = scaled racquet; LR = large racquet; HR = heart rate.

### 3.4 Synthesis of the results

The analysis of all the articles identified different strands of research, into which they were classified, as reflected in Table 5. Although some articles addressed more than one topic and were included in multiple categories, this table provides a global overview of the research on adapted material in tennis, according to the themes identified.

**Table 5:** Classification of the studies according to the research areas identified.

Research area	Studies
Skill acquisition and learning processes	Farrow and Reid (2010), Buszard et al. (2014b), Buszard et al. (2020a), Larson and Guggenheimer (2013), Hammond and Smith (2006), Buszard et al. (2014a), La (2017), Fitzpatrick et al. (2018), Kilit et al. (2023), Prodan and Grosu (2017), Buszard et al. (2020b), Hardoy et al. (2011), Ciuntea (2018), Coldwells and Hare (1994), Buszard et al. (2016b), Fauzi et al. (2021)
Match performance	Kachel et al. (2015), Limpens et al. (2018), Fitzpatrick et al. (2018), Sánchez-Alcaraz (2013), Bayer et al. (2017), Fitzpatrick et al., 2017, Kilit et al. (2024), Schmidhofer et al. (2014), Gimenez-Egido et al., 2020a, Gimenez-Egido et al. (2020b), Gimenez-Egido et al. (2023), Giménez-Egido et al. (2020c), Timmerman et al. (2015)
Analysis of biomechanical variables	Buszard et al. (2020b), Buszard et al. (2020c), Broadbent et al. (2021), Fadier et al. (2023), Touzard et al. (2023)
Influence on psychological variables	Farrow and Reid (2010), Hardoy et al., 2011, Kilit et al. (2024), Gimenez-Egido et al. (2023), Timmerman et al. (2015)
Perceptions and opinions of coaches and professionals	Buszard et al. (2020a), Davies (2019), Cortela et al. (2019), Martínez-Gallego et al. (2022)

## 4 Discussion

The scoping review identified 35 articles that examined equipment and game modifications in tennis, focusing on their influence on relevant factors for beginner players. Several key research lines within this topic were identified and are elaborated below.

### 4.1 Effects on the skill acquisition process

A prominent and frequently addressed topic in the literature on modifying tennis equipment is its impact on the learning process of tennis skills, particularly in the development of technical abilities.

Most of the studies have focused on training methodologies and their role in skill acquisition. The key variables analyzed included stroke technique and performance (Farrow and Reid, 2010; Buszard et al., 2014b; Hammond and Smith, 2006; Buszard et al., 2014a; La, 2017; Fitzpatrick et al., 2018; Kilit et al., 2023). These studies typically examined the phases of the stroke—such as positioning, backswing, impact, and follow-through—comparing performance with scaled and standard equipment. Stroke performance is assessed through variables such as stroke volume, opportunities, and success rates. Buszard et al. (2014b) reported greater technical losses and disruptions with standard equipment compared to scaled equipment. Other studies employed specific skill tests, such as the TSST, as seen in studies by Fitzpatrick et al. (2018) and Kilit et al. (2023).

Notably, research on groundstrokes has shown significant improvements in speed, accuracy, and the relationships between speed, accuracy, and success when using scaled equipment. These adaptations have led to better forehand and backhand performances, with greater precision and power (Larson and Guggenheimer, 2013; Prodan and Grosu, 2017; Buszard et al., 2020b). A significant finding was the reduction in ball speed when using low-compression balls (Larson and Guggenheimer, 2013; Prodan and Grosu, 2017). The slower balls provide players with more time for preparation and positioning, resulting in greater technical efficiency and stroke success.

Studies have also explored broader adaptations, such as comparing mini-tennis to adult tennis, in terms of their effects on basic motor skills. Improvements were observed in hand-eye coordination (Hardoy et al., 2011), agility, and speed (Ciuntea, 2018), as well as in tennis-specific skills such as positioning, stroke technique, and court movement (Coldwells and Hare, 1994; Buszard et al., 2016b). These studies have consistently reported significant improvements and demonstrated that mini-tennis provides a positive transfer to adult gameplay.

In terms of age groups, research in this area has predominantly focused on children aged 6 to 9 years, as scaled adaptations are typically recommended for these age groups. However, more recent studies, such as Kilit et al. (2023), have included beginner adults, demonstrating that the benefits of scaled adaptations are not limited to children. These findings highlight the need for further exploration of the effects of scaled equipment on other populations, including adolescents, older adults, and individuals with functional diversity.

### 4.2 Effects on match performance

Another extensively studied area concerns the impact of equipment modifications in competitive contexts, with a focus on match performance. Research in this domain has often analyzed real or simulated matches, investigating technical-tactical variables and playing styles.

Several studies have examined the effects of equipment modifications on game structure, including point duration and effective playing time. Low-compression balls and reduced court dimensions were found to significantly increase point duration compared to traditional equipment (Sánchez-Alcaraz, 2013; Bayer et al., 2017; Fitzpatrick et al., 2017, 2018; Kilit et al., 2024). These modifications give players more time to react, position themselves, and execute strokes, resulting in longer rallies and more dynamic gameplay. Longer point durations also increase effective playing time, offering more opportunities to practice skills and engage in realistic match scenarios.

Other studies have focused on technical-tactical variables, particularly serve and groundstroke performance. Modifications such as reduced net height and smaller court dimensions have been associated with greater first-serve effectiveness and improved serve placement (Limpens et al., 2018; Bayer et al., 2017; Schmidhofer et al., 2014; Gimenez-Egido et al., 2020a; Gimenez-Egido et al., 2020b; Gimenez-Egido et al., 2023). For groundstrokes, scaled adaptations promote greater symmetry in the use of the forehand and backhand (Fitzpatrick et al., 2018; Fitzpatrick et al., 2017), addressing the common tendency of young players to favor the forehand over the backhand (Farrow and Reid, 2010) and aligning their play more closely with adult tennis.

Reducing net height has been shown to facilitate a more offensive playing style, increasing the frequency of flat, winning shots and net approaches (Giménez-Egido et al., 2020c). In addition, Kachel et al. (2015) reported that green balls resulted in more frequent net play and higher ball speeds. These findings suggest that scaled adaptations facilitate a playing style that mirrors adult tennis, providing players with more opportunities for tactical development.

Despite the consistent focus on technical-tactical variables in competitive scenarios, the studies reviewed varied in methodology, participant age and skill levels, and the specific adaptations implemented. Most studies explored modifications to court size and net height, while others examined balls with varying compression. Further research is needed to investigate the long-term effects of scaled equipment, the transition to standard equipment, and the customization of materials to player characteristics.

### 4.3 Biomechanical variables

Studies examining biomechanical variables associated with scaled tennis equipment have focused on aspects of motor control and performance. Research by Buszard et al. (2020c) and Buszard et al. (2020b) highlighted improvements in stroke precision and distal arm segment control with scaled equipment. These studies observed increased temporal stability and reduced movement variability, along with better coordination between the upper arm and forearm. Methodological differences between the studies included analyses of racket-shoulder distance in relation to task success, control, and impact point in one study and variability in arm segment coordination and flexibility in the other.

Broadbent et al. (2021) expanded the biomechanical focus to include other sports, such as rugby, netball, basketball, and hockey, evaluating the relationship between athletes’ body measurements and sport-specific adaptations. The study noted that adaptations were more acceptable in female competitions than in male ones and that younger athletes (under 9 years old) faced greater challenges with oversized courts and equipment.

In serving, significant improvements in kinematic and kinetic variables were reported (Fadier et al., 2023; Touzard et al., 2023). Fadier et al. (2023) found that reduced court and net dimensions led to higher racket and ball speeds, increased knee extension velocity, and improved trunk flexion in young players. Touzard et al. (2023), while not observing significant differences in racket and ball speeds, reported increased angular velocities in distal arm segments and reduced loads on the shoulder and elbow with scaled rackets compared to standard rackets.

These findings are particularly important for injury prevention in young players aged 8 to 12 years. Scaled equipment reduces joint stress while maintaining, or even enhancing, performance and stroke control. Proper implementation of these adaptations in under-8, under-10, and under-12 competitions is essential for optimizing player development. Future research should investigate injury prevention in other strokes and evaluate scaled equipment’s precision and effectiveness in real-match contexts, as many studies currently focus on controlled conditions.

### 4.4 Psychological variables

Studies examining the psychological effects of equipment modifications have highlighted variables such as practice commitment, enjoyment, and self-efficacy. Scaled equipment has been shown to enhance enjoyment by fostering a more aggressive playing style, which creates a more positive experience for players. Specifically, reduced net height has been associated with increased self-efficacy in beginner players, improving their success rate in serves. This improvement in performance boosts their comfort and enjoyment during practice, ultimately increasing their commitment (Gimenez-Egido et al., 2023; Timmerman et al., 2015). However, these effects were significant only for serves and did not extend to other strokes.

Kilit et al. (2024) observed that playing with green balls resulted in a more positive mood among adult beginner players, whereas using standard balls led to higher tension, fatigue, and frustration, as well as a greater perceived mental effort. Similarly, Farrow and Reid (2010) found that adapted courts and balls increased player engagement compared to standard conditions due to a higher number of opportunities and successful strokes. In contrast, Timmerman et al. (2015) reported that players exhibited more confidence with standard courts and nets, potentially due to unfamiliarity with modified conditions.

The psychological benefits of equipment modifications are especially evident in players with functional diversity. Hardoy et al. (2011) reported reduced anxiety and frustration, as well as improved self-confidence and hand-eye coordination, under adapted conditions in players with intellectual disabilities. These findings underscore the importance of further research focused on individuals with physical and mental disabilities to better understand and expand these benefits.

Insights gained from examining psychological and attitudinal variables are critical for understanding player behavior on the court and enhancing their overall experience. However, psychological variables were often treated as complementary in the studies reviewed, typically included as secondary outcomes alongside technical-tactical or physiological variables. Challenges in this area of research arise from the variability in psychological metrics studied across different articles, which complicates comparisons and interpretations. Moreover, underrepresented groups, such as adults and individuals with functional diversity, require more attention. Future studies should explore psychological and emotional variables more thoroughly across diverse player populations and investigate their interplay with related physiological variables.

### 4.5 Coaches’ and professionals’ perceptions

Four studies in this review examined coaches’ perceptions of the International Tennis Federation’s (ITF) campaigns and the use of modified equipment, consistently highlighting a positive impact on children’s learning. Hammond and Smith (2006) conducted interviews with coaches, who unanimously praised low-compression balls for improving beginner players’ control and technique.

Using a qualitative approach, Davies (2019) found that coaches preferred the Constraints-Led Approach methodology and valued scaled equipment programs. They emphasized the importance of aligning equipment with players’ skills rather than biological age and advocated for cooperative, varied teaching methods. Cortela et al. (2019) examined the transition from green to yellow balls, revealing that individual coaches relied on specific criteria, such as impact height and grip, with the commencement of competitive play being a decisive factor.

From a quantitative perspective, Buszard et al. (2020a) evaluated professionals’ opinions on the Tennis 10s, Tennis Xpress, and ITF Play and Stay campaigns and regulatory changes. They concluded that these initiatives effectively increased tennis participation and improved key skills, particularly the forehand and backhand. Similarly, Martínez-Gallego et al. (2022) focused on the ITF Play and Stay campaign and regulatory changes in under-10 competitions. They confirmed that coaches were familiar with and applied these standards during training and competitions and had access to the necessary equipment for implementation. The study concluded that the campaign positively influenced participation and learning among beginner players.

These findings provide essential insights for federations and organizations managing grassroots and school tennis programs, highlighting the effectiveness of these campaigns and coaches’ satisfaction with their implementation. Nonetheless, future research could expand this area of inquiry by employing quantitative, qualitative, and mixed methods to deepen the understanding of coaches’ perspectives. In addition, extending the analysis to include federations and organizing bodies could offer a broader perspective on the adoption and impact of these initiatives.

## 5 Limitations

As a scoping review, this study prioritized identifying general themes and trends over in-depth analyses of causal relationships or specific effects. One limitation is the representativeness of the results, as the inclusion criteria depended on the availability of studies in certain languages or databases. Furthermore, scoping reviews are not designed to answer specific research questions or synthesize evidence quantitatively, necessitating systematic reviews or meta-analyses to validate robust conclusions.

## 6 Future research directions

Based on the results from this review, future research should focus on real-game contexts, delve deeper into biomechanical aspects for injury prevention and development optimization in children, and prioritize the study of psychological variables. Including underrepresented groups, such as adults and individuals with functional diversity, would provide valuable insights. Furthermore, examining gender- and age-related differences in greater detail is recommended.

### 6.1 Practical applications

These findings hold relevance for federations, clubs, and coaches, who can standardize the use of scaled equipment in training programs and competitions to facilitate beginner players’ technical progression, foster greater adherence to the sport, and improve retention rates. In under-10 competitions, events organized by federations should universalize these adaptations in their regulations to prevent injuries and promote safe tennis practice from an early age. Insights into the game structure can help clubs organize coaching programs for players of all ages. Coaches can facilitate skill acquisition by planning training sessions for beginner players that offer opportunities to focus on strategic learning and decision-making. Finally, integrating this approach into coach education programs worldwide, accompanied by certifications issued by federations, would help standardize these methodologies among professionals.

## 7 Conclusion

This scoping review aimed to identify and categorize existing research on scaled equipment in tennis and summarize the most relevant findings within each area, providing a preliminary map of the evidence without assessing the quality or validity of individual studies. The main findings suggested that scaled adaptations improve stroke control and precision, facilitate the learning of basic techniques, and promote a more efficient and tactical playing style. In addition, they reduce joint stress, minimize injury risk, and enhance satisfaction, self-efficacy, and commitment during practice in young players. Coaches positively regard scaled equipment for its effectiveness in teaching processes.

## Data Availability

The raw data supporting the conclusions of this article will be made available by the authors, without undue reservation.
