# Supplementary material for: Exploring the impact of equipment modifications on novice tennis players: a scoping review
Source: Front Psychol. 2025 Feb 26;16:1536427. doi: 10.3389/fpsyg.2025.1536427 (PMC11897481; doi:10.3389/fpsyg.2025.1536427)
Supplement: Supplementary file 1 [file Data_Sheet_1.pdf]

Reference	Topic	Sample & methods	Results
Coldwells and Hare (1994)	To observe the transfer of skills acquired in mini tennis (MT) to the real tennis game.	N=16 (7-10 years). EG (10h MT, 10h real tennis) and CG (20h real tennis).<br>N=14 (7-10 years). EG (4h MT, 4h real tennis) and CG (8h real tennis).	The experimental group demonstrated greater skills after training than the control group in both studies. Mini tennis had a positive transfer in faster skill acquisition to real tennis.
Hammond and Smith (2006)	To investigate the effectiveness of low compression balls (LCB) on the learning of skills.	N=14 (5-11 years). Experimental group (LCB) and control group (standard balls). 8 training sessions.	There were no significant differences between the groups, although LCB group improved the most in technique, point duration and hitting opportunities.
Farrow and Reid (2010)	To determine the effect of adapted equipment and tasks on skill acquisition in young tennis players.	N=23 (8 years) with limited or no previous tennis experience. 5 weeks of training in one of the 4 conditions.	The groups practicing in adapted conditions significantly improved in rally volume, forehand technique, backhand technique, number of chances, success, enjoyment and commitment to practice.
Hardoy et al. (2011)	To evaluate the effectiveness of a mini tennis program as a therapeutic aid in the rehabilitation of players with intellectual disabilities.	N=24 (18-40 years old), experimental group and control group. 6 months, sessions of 3h/2 times a week.	A decrease in the anxiety scale and improvements in hand-eye coordination was observed in the experimental group compared to the control group. No significant differences were found in body perception.
Larson and Guggenheimer (2013)	Determine the effects of modifying the court and the ball on the forehand stroke.	N=8 (7-9 years with experience).	With LCB and reduced courts, significantly higher speed-accuracy-success scores were obtained, increasing the chances of quality hitting.
Sánchez-Alcaraz (2013)	To analyze the temporal structure and actions of competitive play in young players.	N=8 (8 years with experience). 16 matches analyzed (8 orange, 8 yellow).	Playing in the reduced court and orange ball produced longer points, a greater number of strokes, less unforced errors and more winners with significant difference compared to the conventional.
Buszard et al. (2014a)	To examine the influence of different racquet sizes and ball compressions in young tennis players.	N=80 (6-8 years without experience). Performed a task with 3 racquet sizes and 3 types of ball.	The combination of small racquet and red ball produced higher hitting performance. Red ball had the best results combined with small/medium racquets.

Buszard et al. (2014b)	To observe if with adapted material they showed less conscious involvement than standard material when learning a tennis motor task.	N=40 (9-11 years old), one expert group and other less skilled. Performed a task with 2 types of material and their working memory and motor skills were measured.	Less skilled group performed significantly worse when using normal-sized equipment. The more skilled children only failed using normal equipment. They stepped forward, swung the racquet from bottom to top and made a correct impact with the ball more often with adapted equipment.
Schmidhofer, et al. (2014)	Compare the structure of play between ATP tennis and the three levels of Tennis 10s, to quantify the differences.	N=87 (67 elite players, 20 children in U9, U10, U12).	In elite players, more aces and % of first serves in were observed, as well as less % of points won with the rest, lower ICT and shorter duration of rallies. In U9, these values were better in a reduced court.
Kachel et al. (2015)	To examine adapted equipment on elite junior players in a competition.	N=20 (10 years). Played two matches, one with yellow ball and the other with green ball.	Modified ball: the rally speed was faster; more shots hit at a comfortable height and more shots hit at the net. With the yellow ball more "high" shots were hit.
Timmerman et al. (2015)	To examine the modification of court size and net height in tennis players based on adult game speed.	N=16 (9 years). Matches in 4 conditions: scaled court and net, scaled net-standard court, standard net-scaled court, conventional court and net.	Scaled net: more winners, forced errors, shots at a comfortable height, which meant that players adopted a more aggressive style of play. The adapted court and net resulted in a faster game comparable to the adult game.
Buszard et al. (2016b)	To examine the influence of racquet adaptation on skill acquisition in elementary school children.	N=46 (PE Grade 1 and 2) a group with small racquet (SR), and another with large racquet (LR). 30min x 5 weeks with red ball.	The SR group improved significantly more in forehand and backhand than the LR. The small racquet group seemed to improve transfer to the large racquet, but not the other way around.
Bayer, Ebert and Leser (2017)	Examine if the Lima court is a solution to the undesired effects of the transition from the Orange to Green court.	N=24 (9-10 years) 12 matches on green court and 12 on lime court.	The structure of play on Lima court is more like the adult game than on green court. Lima court: more points won with the 2nd serve and the time between contacts was closer to adult tennis. More forced errors and winners were observed in Lima.
Fitzpatrick, et al. (2017)	To determine whether court and ball dimensions affect playing behavior in children's games.	N=48 divided into 4 groups (7, 8, 9 and 13 years). Thirty-five games were analyzed.	Points duration and number of forehands was in decline as the age groups progressed, but there was more variability of play. Younger group: % of first serves and number of strokes was higher.
Prodan and Grosu (2017)	To investigate the influence of different racquet sizes and ball	N=20 (8-9 years), experimental group with red,	The accuracy-speed variable was much higher with adapted equipment. Low-pressure balls give children

	compression on backhand groundstrokes accuracy.	orange and green balls and control group.	more opportunity to make successful hits, with more accuracy and speed.
Sanz (2017)	To compare the traditional methodology to the alternative and validate the effect of the adapted material in beginners.	N=100 (U10) 2 experimental groups (7/8 years orange ball; 9/10 years green ball) and 2 control groups.	Greater improvements were observed in the experimental groups than in the control groups, both at the technical and execution level, as well as at the tactical level.
Ciuntea (2018)	To analyze the influence of the Tennis10 method in the development of basic motor skills in children.	N=56 (7-9 years), experimental and control group. 8 months intervention 2 sessions of 1h/week.	In all tests, the experimental group improved more than the control group. Children up to 10 years of age benefit from the Tennis10 method because of the gradual resizing of the materials.
Fitzpatrick et al. (2018)	To investigate the effects of adaptations on match behavior and performance in tennis skills tests (backhand).	N=16 (7 years) experimental and control group. 8 weeks intervention 1h/week	Experimental group presented better symmetry in strokes than in the pretest. Higher success rates in backhand, an improvement in rally and greater technical competence were observed.
Limpens et al. (2018)	To examine reductions in net height on match performance in U10 players.	N=16 (9 years) 4 games each pair in the 4 heights of the net.	Lower net height improves serving performance, facilitates more aggressive and faster game, more groundstrokes and winners; decreases rally duration.
Cortela et al. (2019)	Describe the transition process from green ball to yellow ball in Brazilian clubs.	N=14 Brazilian club coordinators with previous experience in tennis initiation programs.	It was not possible to clearly identify criteria for the process. It appears that the “clinical eye” of the trainer is the main parameter for determining when the transition occurs.
Davies (2019)	Perspectives of tennis coaches on the use of adapted equipment.	N=20 coaches with experience in adapted equipment were interviewed.	It is imperative to ensure adequate skill acquisition before moving to the next level, and transitions should not be made by age but by skill level.
Giménez-Egido et al. (2020c)	To know the impact of a modified competition on the strokes of U-10 players using a smart sensor.	N=20 (U10) 4 matches each in different conditions of net height and court dimensions.	In modified competition, increased the number of flat shots and was a more offensive style of play, but less variability of shots was observed.
Buszard et al. (2020c)	Determine whether shoulder-shoe distance is a determining variable and evaluate the hand-shoe segment.	N=21 (6-9 years) one group with adapted equipment and one normal group. 40 attempts of forehand strokes.	The distance between shoulder and racquet is a determinant variable of performance regardless of the equipment used. Players with adapted equipment have more control in the hand-racquet distance.

Buszard et al. (2020a)	Analyze the perceptions of coaches in implementing a modified tennis campaign on participation and skill development.	N=114 (35 key figures from different national tennis associations and 79 coaches)	Majority of the participants perceived that Play and Stay is associated with increased and sustained participation and promotes skill development. They also supported that the campaign has a positive effect on attitude in children and adults.
Buszard et al. (2020b)	Identify how material adaptation facilitates coordination and movement variability when performing a precision tennis hitting task.	N=25 right-handed hitting task to a target in 2 conditions: with adapted equipment and standard equipment.	Adapted materials allowed greater accuracy in the stroke, greater temporal stability of the swing and a coupling between the variability of the upper arm and forearm movements; compared to full-size equipment, with which this did not occur.
Gimenez-Egido et al. (2020a)	To compare two competition formats adapted on the serve in U-10 players.	N=20 (U10) Serves of 80 matches were analyzed, half of them in U10 conditions, the other in adapted conditions.	In the modified competition, there were a higher number of effective serves, direct serves and unreturned serves. Reducing the net height and court dimensions improved serving performance.
Gimenez-Egido et al. (2020b)	To analyze the effects of modifying the height of the net and the dimensions of the court on technical-tactical variables in U-10 players.	N=20 (U10) 40 matches in 2 conditions, half of them in ITF U10 conditions (GC), and the other half in adapted conditions (MC).	MC: greater variability and opportunity for different strokes and patterns, more hits close to the net, with a more offensive character. Fewer 2nd serves were produced, with greater effectiveness of the serve in adapted court.
Broadbent et al. (2021)	Explore the use of pi ratios as a basis for establishing adaptations to Australia's most popular junior ball sports.	Federation guidelines and junior modifications were compared with height and maturation from children's databases. Ratios were calculated.	Most Australian children compete at larger sizes than would be appropriate for their ability, especially in the younger age groups. Female competitions were better suited to physical conditions than male competitions. The U-15 age group is the only one in which all sports are in an acceptable range.
Fauzi et al. (2021)	To determine the effect of a mini-tennis training model on forehand groundstroke.	N=44 (6-8 years), experimental group (mini tennis training) and control group (conventional training).	Significant differences were found between pre-test and post-test of the control group and experimental group. The experimental group obtained better results in post-test than the control group.
Martínez-Gallego et al. (2022)	Discover the perspectives of national federation leaders on the ITF's Play and Stay campaign.	N=35 experts from different national federations related to training programs answered a questionnaire.	Most of the experts were aware of the rule change and applied it, agreed on the benefits and availability of materials to enable it. There was strong agreement that it has a positive impact on U-10 players.
Fadier et al. (2023)	To determine whether the reduction of serve distance	N=10 (9-12 years) 3 serves with green ball from the 3	Reducing court dimensions helps to improve hitting performance. In red conditions: more powerful and

	and net height affects the biomechanics and performance.	distances (red, orange & green measures). Biomechanical variables were measured.	faster serves were produced. Maximum angular velocities of trunk and knee flexion also improved which favors the development of the serve.
Kilit et al. (2023)	To evaluate the effects of ball compression and court size on skill learning in adult tennis players.	N= 24 university students with no experience. 1 week of pre-test, 6 weeks of training and 1 week of post-test (specific tennis skills test).	The LCB group scored higher in accuracy, groundstrokes, rally performance, technique control, speed and direction. Adapted equipment provides greater stability in strokes, increased pace of play, and a positive impact on skill learning in adults.
Touzard et al. (2023)	To evaluate the effects of racquet size on serve biomechanics and performance variables in young tennis players.	N= 9 (10 years) with markers performed 3 effective flat serves with 3 different sized racquets. The trajectories of the serves and kinematic variables were analyzed.	Racquet size did not influence ball speed, racquet head speed, or % of effective serves. It influenced shoulder and elbow loads, increasing the risk of injury to the dominant upper limb. With small racquets: higher distal angular velocities, with conventional racquets: higher proximal velocities.
Gimenez-Egido et al. (2023)	To evaluate the effect of reducing net height and court size on self-efficacy and hitting efficiency of U-10 players in a real game context.	N= 20 Perceived Physical Ability, Self-Efficacy in Tennis with questionnaires, and technical-tactical actions in standard and adapted conditions were measured.	There were no significant differences in self-efficacy between the two playing conditions. They show higher self-efficacy and service efficiency when net height and court size were reduced, as lower net height and court size increased the percentage of successful serves.
Kilit et al. (2024)	To compare the effects of playing with low compression balls and standard balls on psychophysiological responses and match characteristics in adult tennis players.	N= 24 (18-34 years). 2 matches (green ball and standard ball). HR, % HR, game characteristics, PE, enjoyment, mental effort and mood profile were measured.	Green ball play was associated with higher psychophysiological responses: higher HR, % HR and enjoyment of the activity. It also resulted in greater game characteristics such as longer rallies and a more controlled pace of play. The standard ball caused higher perceived exertion and less positive mood state

Extended version of the table 4, article's included main characteristics.
